# Rer1-mediated quality control system is required for neural stem cell maintenance during cerebral cortex development

**DOI:** 10.1371/journal.pgen.1007647

**Published:** 2018-09-27

**Authors:** Taichi Hara, Ikuko Maejima, Tomoko Akuzawa, Rika Hirai, Hisae Kobayashi, Satoshi Tsukamoto, Mika Tsunoda, Aguri Ono, Shota Yamakoshi, Satoshi Oikawa, Ken Sato

**Affiliations:** 1 Laboratory of Molecular Traffic, Institute for Molecular and Cellular Regulation, Gunma University, Maebashi, Gunma, Japan; 2 Laboratory of Cellular Regulation, Faculty of Human Sciences, Waseda University, Mikajima, Tokorozawa, Saitama, Japan; 3 Laboratory Animal and Genome Sciences Section, National Institute of Radiological Sciences, National Institutes for Quantum and Radiological Science and Technology, Anagawa, Inage-ku, Chiba, Japan; Stanford University School of Medicine, UNITED STATES

## Abstract

Rer1 is a retrieval receptor for endoplasmic reticulum (ER) retention of various ER membrane proteins and unassembled or immature components of membrane protein complexes. However, its physiological functions during mammalian development remain unclear. This study aimed to investigate the role of Rer1-mediated quality control system in mammalian development. We show that Rer1 is required for the sufficient cell surface expression and activity of γ-secretase complex, which modulates Notch signaling during mouse cerebral cortex development. When Rer1 was depleted in the mouse cerebral cortex, the number of neural stem cells decreased significantly, and malformation of the cerebral cortex was observed. Rer1 loss reduced γ-secretase activity and downregulated Notch signaling in the developing cerebral cortex. In Rer1-deficient cells, a subpopulation of γ-secretase complexes and components was transported to and degraded in lysosomes, thereby significantly reducing the amount of γ-secretase complex on the cell surface. These results suggest that Rer1 maintains Notch signaling by maintaining sufficient expression of the γ-secretase complex on the cell surface and regulating neural stem cell maintenance during cerebral cortex development.

## Author summary

We showed that Rer1 functions as an early-Golgi quality control pathway that maintains γ-secretase activity by maintaining sufficient cell surface expression of γ-secretase complex during cerebral cortex development, thereby modulating Notch signaling.

## Introduction

Newly synthesized proteins are primarily assessed by the ER quality control system for correct folding, modification, and complex formation. Nevertheless, some immature or misfolded proteins escape from the ER to post-ER compartments. However, they are recognized by several retrieval receptors at the early-Golgi compartment and are hence returned to the ER. The latter mechanism is referred to as the “early-Golgi quality control system”.

Rer1 is a well-conserved early-Golgi membrane protein in eukaryotes, which plays important roles in the early-Golgi quality control system. Rer1p was originally identified in yeast as a receptor for the retrieval of ER membrane proteins from the early-Golgi to the ER via the COPI-mediated retrograde transport pathway [1–4]. Recently, the roles of Rer1 have been extended to the ER retention of immature or misfolded mutant membrane proteins related to some ER misfolding diseases [5, 6]. Importantly, Rer1 also modulates the formation of multimeric membrane protein complexes by retaining unassembled components in the ER. In yeast, Rer1p recognizes an unassembled iron-transporter subunit Fet3p and retains it in the ER until Fet3p forms an appropriate complex with its partner, Ftr1p [7]. These observations suggest that Rer1 functions as a sorting chaperone to support the formation of multimeric membrane protein complexes [4, 7].

γ-Secretase is a multimeric membrane protein complex, which comprises presenilin-1 (PS1), nicastrin (NCT), anterior pharynx-1 (APH-1), and presenilin enhancer γ-secretase subunit (also known as PEN2) [8–13]. Unassembled subunits of the γ-secretase complex are retained or degraded in the ER [14]. Only properly assembled γ-secretase is transported to post-Golgi compartments and functions as an intramembrane aspartyl protease, which cleaves type I transmembrane proteins such as amyloid precursor protein (APP) and Notch [15–17]. In this process, Rer1 binds unassembled NCT and/or PEN2 and retains them in the ER [18, 19]. Since siRNA-mediated knockdown of Rer1 enhances complex formation and the activity of the γ-secretase complex in cultured cells, Rer1 has been considered to negatively regulate γ-secretase function via competition with APH-1 for NCT binding [19–21].

The present study aimed to investigate the role of the Rer1-mediated quality control system in mammalian development. First, we generated Rer1 knockout mice and determined whether it is essential in early mouse development. Furthermore, we examined the role of Rer1 in mouse cerebral cortex development. Our study hence reveals the primary role of Rer1 in Notch signaling in the cerebral cortex development and in the regulation of the neural stem cell population.

## Results

### Rer1 is required for mouse early development

To examine the physiological roles of Rer1 in mammalian development, we generated a knockout mouse using an embryonic stem (ES) cell line (EUCOMM), in which a reversible gene-trap cassette, FlipRosaβgeo, is inserted in the intron 1 of *Rer1* (S1A and S1B Fig). We first generated *Rer1* heterozygous gene-trap (*Rer1*^*+/trap*^) mice, using this ES cell line. We confirmed a single gene-trap cassette insertion into the *Rer1* locus in *Rer1*^*+/trap*^ mice via Southern blot analysis (S1A and S1C Fig). Although the *Rer1*^*+/trap*^ mice showed normal gross morphology and fertility, these mice were 10–20% lighter than *Rer1*^*+/+*^ mice (S1A and S1B Fig). The protein level of Rer1 was reduced in *Rer1*^*+/trap*^ mice (S2C Fig), suggesting that heterozygous loss of Rer1 results in haploinsufficiency in body size (S2A–S2C Fig). Furthermore, we attempted to generate Rer1-homozygous gene-trap mice (hereafter *Rer1*^*trap/trap*^) by intercrossing *Rer1*^*+/trap*^ mice. However, *Rer1*^*trap/trap*^ mice were embryonic lethal, as reported previously [22], indicating that Rer1 plays an essential role in mouse early development (S1D Fig). To circumvent the developmental lethality of Rer1 deficiency, we crossed *Rer1*^*+/trap*^ mice with *CAG-NLS-FLPe* transgenic mice (S3A–S3C Fig). *Rer1*^*inv-trap*^ homozygous mice were born at the predicted Mendelian ratio, indicating that the embryonic lethality by Rer1 gene inactivation using the gene trap cassette was primarily canceled by flipping it to the noncoding strand *in vivo*.

### Rer1 is required for normal cerebral cortex development

Since Rer1 is involved in the formation of the γ-secretase complex, we focused on the roles of Rer1 in the mouse brain development. To inhibit Rer1 expression in the developing cerebral cortex by reinverting the inverted trap cassette in the forebrain of *Rer1*^*inv-trap*^ homozygous mice, we crossed *Rer1*^*inv-trap*^ homozygous mice with *Emx1-Cre* transgenic mice, which express a Cre recombinase only in the forebrain of embryos and the cerebral cortex of adult mice [23]. The resulting *Rer1*^*+/inv-trap*^; *Emx-Cre* mice were crossed with *Rer1*^*inv-trap*^ homozygous mice to generate forebrain-specific Rer1 conditional knockout mice (*Rer1*^*inv-trap/ inv-trap*^; *Emx-Cre* mice). The forebrain-specific Rer1 conditional knockout (Rer1 cKO) mice were born at the Mendelian ratio. However, half of forebrain-specific Rer1 cKO mice died soon postpartum. The remaining forebrain-specific Rer1 cKO mice survived for more than one year.

**Fig 1:**
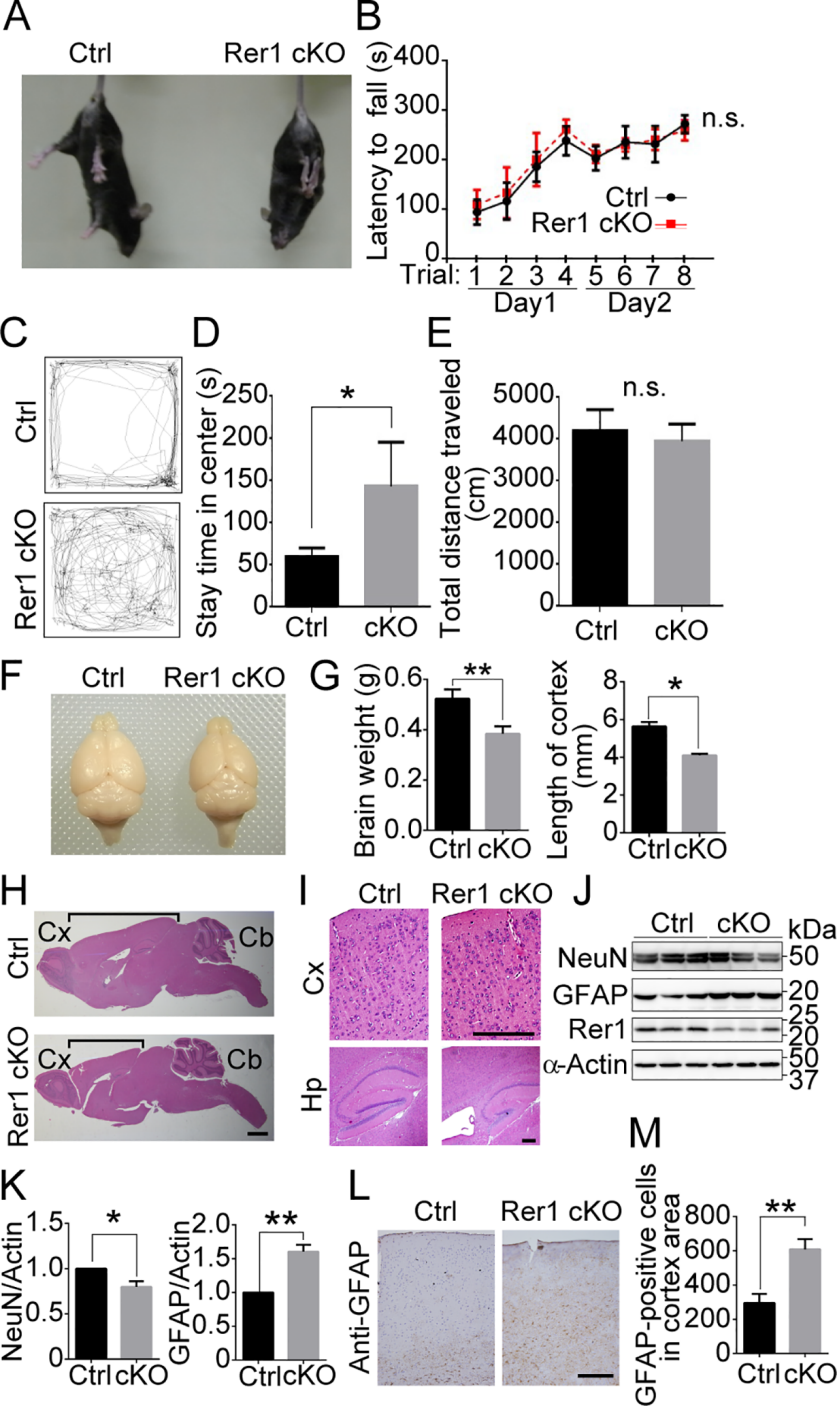
Rer1-deficiency in the mouse forebrain results in cerebral degeneration. (A) Abnormal limb-clasping reflexes in forebrain-specific Rer1 cKO mice. Control (*Rer1*^*+/inv-trap*^) and forebrain-specific Rer1 cKO (*Rer1*^*inv-trap/inv-trap*^; *Emx-Cre*) mice were suspended by the tail. The forebrain-specific Rer1 cKO mice held and tightened their limbs. (B) Rotarod test of control (*Rer1*^*+/inv-trap*^, *Rer1*^*inv-trap/inv-trap*^, *Rer1*^*+/inv-trap*^; Emx-Cre; n = 7) and forebrain-specific Rer1 cKO (*Rer1*^*inv-trap/inv-trap*^; *Emx-Cre*; n = 5) male mice at 3 months of age. Mice were placed on a rotating rod and the time spent on the rod was measured. Data are shown as the mean ± standard error of the mean (SEM). n.s.: not significant. (C) Abnormal open field behavior in forebrain-specific Rer1 cKO mice. Traces show representative exploratory behavior in an open field for control (*Rer1*^*+/inv-trap*^) and forebrain-specific Rer1 cKO (*Rer1*^*inv-trap/inv-trap*^; *Emx-Cre*) mice. (D, E) Quantitative analysis of time spent in the center (D) and total distance traveled (E) for 3-month-old control (*Rer1*^*+/inv-trap*^, *Rer1*^*inv-trap/inv-trap*^, *Rer1*^*+/inv-trap*^; *Emx-Cre*; n = 7) and forebrain-specific Rer1 cKO (*Rer1*^*inv-trap/inv-trap*^; *Emx-Cre*; n = 5) male mice. *P < 0.05 (Student *t*-test). n.s.: not significant. (F) Dorsal views of whole-mount brains from 5-month-old male control (*Rer1*^*+/inv-trap*^) and forebrain-specific Rer1 cKO (*Rer1*^*inv-trap/inv-trap*^; *Emx-Cre*) male mice. (G) The brain weight (left graph) or the length of cortex (right graph) of 5-month-old male control (*Rer1*^*+/inv-trap*^) and forebrain-specific Rer1 cKO (*Rer1*^*inv-trap/inv-trap*^; *Emx-Cre*) male mice. Error bars indicate the standard error of the mean (SEM) of three mice. Data are shown as the mean ± standard error of the mean (SEM). P*<0.05, **P < 0.01 (Student *t*-test). (H, I) Hematoxylin and eosin (H&E)-stained sagittal sections from 5-month-old male control (*Rer1*^*+/inv-trap*^) and Rer1cKO (*Rer1*^*inv-trap/inv-trap*^; *Emx-Cre*) male mice. (H) The black line shows the length of the cerebral cortex. Shortening of the cerebrum was observed in Rer1 cKO mice. Cx: Cortex, Cb: Cerebellum. Scale bar, 1 mm. (I) Enlarged images of H&E-stained sections of mediolateral cerebral cortex. Cortical thinning and hippocampal morphogenesis anomalies were observed in forebrain-specific Rer1 cKO mice. Cx: Cerebral cortex, Hp, Hippocampus. Scale bar, 200 μm. (J) Neuron loss and gliosis occur in forebrain-specific Rer1 cKO mice. Homogenates of dissected cerebral cortex from control or forebrain-specific Rer1 cKO mice at 5 weeks were immunoblotted with anti-NeuN (neuronal marker) and anti-GFAP (astrocyte marker) antibodies. An anti-actin antibody was used as a loading control. An anti-Rer1 antibody was used to confirm the Rer1 level. (K) Quantitative analysis of the levels of NeuN and GFAP shown in panel J. Graphs show fold changes of NeuN or GFAP (signal intensity of targets normalized to that of actin) from forebrain-specific Rer1 cKO (cKO, gray bar) mice relative to control (Ctrl, black bar) mice. Error bars indicate the SEM of three mice. *P < 0.05, **P < 0.01 (Student *t*-test). (L) Gliosis in forebrain-specific Rer1 cKO mice. Immunohistochemistry using an anti-GFAP antibody on cerebral cortex from 5-month-old control (*Rer1*^*+/inv-trap*^) and forebrain-specific Rer1 cKO (*Rer1*^*inv-trap/inv-trap*^; *Emx-Cre*) male mice. Scale bar, 200 μm. (M) Graph show quantitative analysis of GFAP-positive cells in control (Ctrl, black bar) or forebrain-specific Rer1 cKO (cKO, gray bar) cortex. Error bars indicate the standard error of the mean (SEM) of six mice. **P < 0.01 (Student *t*-test).

The forebrain-specific Rer1 cKO mice showed limb-clasping reflexes when suspended by their tails, whereas control mice (*Rer1*^*+/inv-trap*^, *Rer1*^*inv-trap /inv-trap*^, *Rer1*^*+/inv-trap*^; *Emx-Cre*) extended their limbs (Fig 1A). In the rotarod test to assess motor coordination, control and forebrain-specific Rer1 cKO mice showed a similar behavior on days 1 and 2, suggesting their normal motor function and learning (Fig 1B). We also examined anxiety and general locomotor activity levels of Rer1 cKO mice via the open field test (Fig 1C). Interestingly, forebrain-specific Rer1 cKO mice spent more time than control mice around the center of the field, suggesting that Rer1 loss in the forebrain reduces anxiety (Fig 1D). However, total distance traveled by control and forebrain-specific Rer1 cKO mice were comparable (Fig 1E), indicating that locomotor activity of forebrain-specific Rer1 cKO mice was normal.

The brain weight and size of cortex from the forebrain-specific Rer1 cKO brain appeared 20–30% smaller than that of control mice (Fig 1F and 1G). We stained sagittal sections of the brains from 5-month-old control and forebrain-specific Rer1 cKO mice with hematoxylin and eosin (H&E) and found that forebrain-specific Rer1 cKO mice had a smaller cerebral size than control mice (Fig 1H). The forebrain-specific Rer1 cKO mice had a thinner cerebral cortex with a lower cell density and a smaller hippocampus than control mice (Fig 1I). Since neurodegeneration is often accompanied by inflammatory responses such as gliosis, we analyzed the neuronal and astrocyte populations in control and forebrain-specific Rer1 cKO mice brains. We first prepared the lysates of the cerebral cortex from 5-week-old control and Rer1 cKO mice and examined the protein levels of a neuronal marker, NeuN, and an astrocyte marker, GFAP via western blot analysis (Fig 1J). NeuN protein levels in forebrain-specific Rer1 cKO mice was slightly but significantly lower than that of control mice (*P* < 0.05) (Fig 1K). In contrast, GFAP protein levels apparently increased in forebrain-specific Rer1 cKO mice at 5 weeks postpartum, compared with that in control mice (*P* < 0.01) (Fig 1K). We also examined the cerebral cortex of 5-month-old control and forebrain-specific Rer1 cKO mice via immunohistochemical staining with anti-GFAP antibody (Fig 1L). GFAP immunoreactivity increased by approximately 2 folds in the cerebral cortex of forebrain-specific Rer1 cKO mice (*P* < 0.01) (Fig 1M), suggesting that Rer1 loss causes prominent gliosis in the mouse brain.

### Rer1 is important for the generation of layer (II–IV) neurons in the cerebral cortex

**Fig 2:**
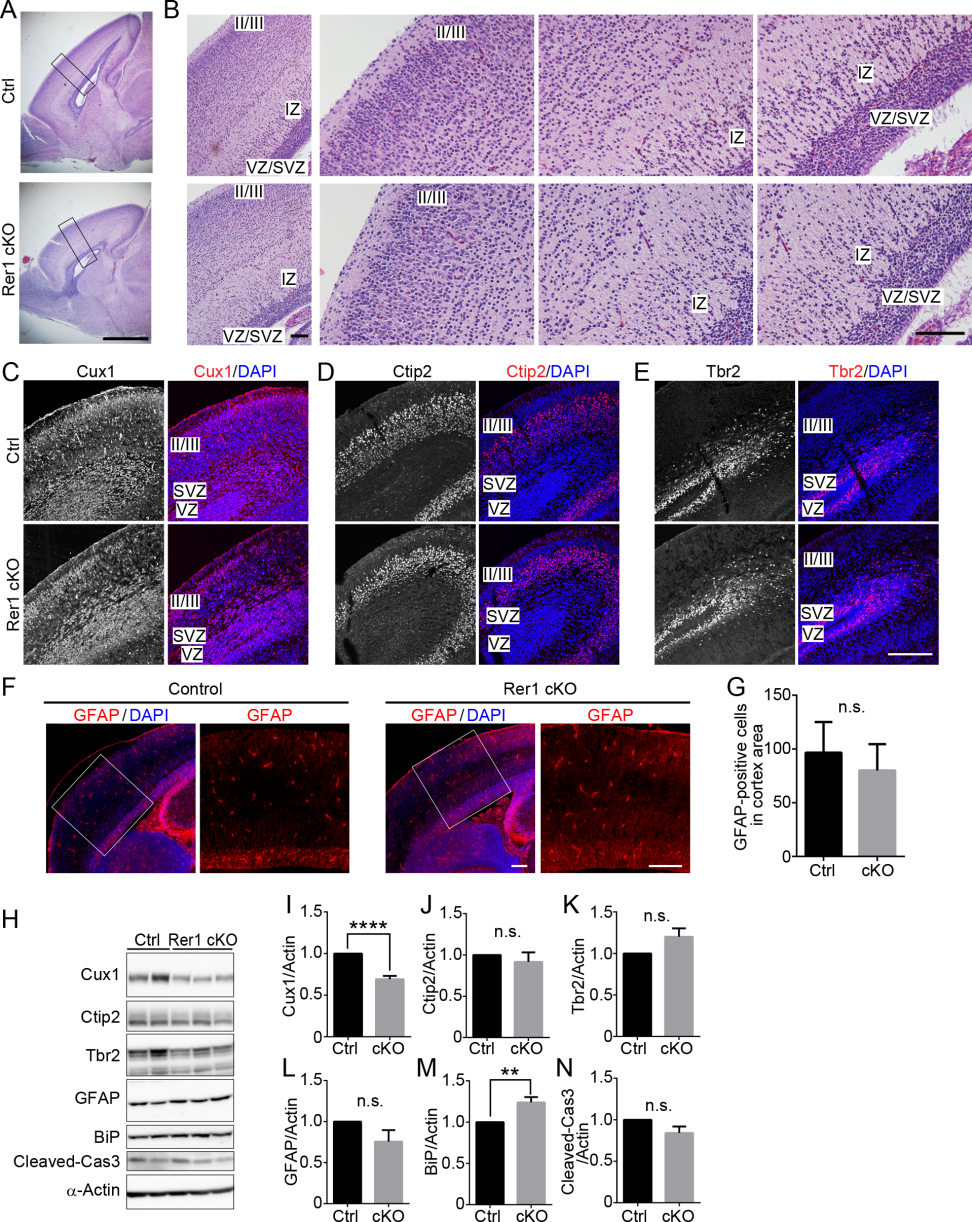
Rer1 deficiency causes cerebral cortex malformations during cortical development. (A) Hematoxylin and eosin (H&E)-stained sagittal cerebral sections of P0 brains from male control (*Rer1*^*+/inv-trap*^) and forebrain-specific Rer1 cKO mice (*Rer1*^*inv-trap/inv-trap*^; *Emx-Cre*). Scale bar, 1 mm. (B) Enlarged views of H&E-stained sections of cerebral cortex in panel A. Small pyramidal neurons and stellate neurons were relatively sparse in layer II/III of the forebrain-specific Rer1cKO brain. IZ: intermediate zone, SVZ: subventricular zone, VZ: ventricular zone. Scale bar, 100 μm. (C-E) Immunohistochemistry of layer markers (red) in the cortex of control (*Rer1*^*+/inv-trap*^) and Rer1 cKO (*Rer1*^*inv-trap/inv-trap*^; *Emx-Cre*) mice on E17.5. Cryosections were immunostained with anti-Cux1 (layers II-IV; C), anti-Ctip2 (layer V; D), and anti-Tbr2 (intermediate progenitors in the SVZ; E) antibodies. Nuclei were stained with DAPI (blue). Scale bar, 200 μm. (F) Immunohistochemistry of GFAP (red) in the cerebral cortex of control and forebrain-specific Rer1 cKO mice on E18.5. Cryosections were immunostained with an anti-GFAP antibody (red). Nuclei were stained with DAPI (blue). Scale bar, 200 μm. (G) Graph show quantitative analysis of GFAP-positive cells in control (Ctrl, black bar) or forebrain-specific Rer1 cKO (cKO, gray bar) cortex on E18.5. Error bars indicate the standard error of the mean (SEM) of six mice. n.s.: not significant (Student *t*-test). (H) Immunoblot analysis of Cux1, Ctip2, Tbr2, GFAP, BiP, and cleaved Caspase-3 in the cerebral cortex of control and forebrain-specific Rer1 cKO mice. Homogenates of dissected cerebral cortices from control and Rer1 cKO mice on E18.5 were immunoblotted with the indicated antibodies for markers of upper layer neurons (Cux1), deep layer neurons (Ctip2), intermediate progenitors (Tbr2), apoptosis (cleaved Caspase-3), astrocyte (GFAP), and ER stress (BiP). An anti-α-Actin antibody was used as a loading control. (I-N) Quantification of immunoblot analysis of Cux1 (I), Ctip2 (J), Tbr2 (K), GFAP (L), BiP (M), and cleaved Caspase-3 (N) as shown in panel H. Graphs show fold changes of values (signal intensity of each marker normalized to that of actin) from forebrain-specific Rer1 cKO (cKO, gray bar) mice relative to control (Ctrl, black bar) mice. Error bars indicate the standard error of the mean (SEM) of six mice. **P < 0.01, ****P < 0.0001 (Student *t*-test). n.s.: not significant.

On postnatal day 0 (P0), brain size was comparable between forebrain-specific Rer1 cKO mice and controls (*Rer1*^*+/inv-trap*^, *Rer1*^*inv-trap/inv-trap*^, *Rer1*^*+/inv-trap*^; *Emx-Cre*) (Fig 2A). However, the cellularity of the upper cortical layer (especially layer II/III) was reduced in the forebrain-specific Rer1 cKO brain at P0 (Fig 2B). We also evaluated cortical malformations via immunostaining, using antibodies against layer markers, Cux1 (layers II-IV), Ctip2 (layer V), and Tbr2 (intermediate progenitors in the subventricular zone, SVZ). The Cux1 signal decreased in the forebrain-specific Rer1 cKO brain (Fig 2C). In contrast, Ctip2- and Tbr2-positive neurons were observed to a similar extent in control and forebrain-specific Rer1 cKO cerebral cortex (Fig 2D and 2E). We also examined GFAP expression in the cerebral cortex of these mice on E18.5 via immunohistochemistry (IHC) (Fig 2F) and found no significant difference between control and the forebrain-specific Rer1 cKO brain on E18.5 (Fig 2G), suggesting that switching from neurogenesis to gliogenesis normally takes place during the brain development. We further conducted western blot analysis of neuronal markers and markers for gliosis, ER stress, and apoptosis in cerebral lysates on E18.5 and quantitated their levels (Fig 2H). Cux1 level was significantly reduced in the forebrain-specific Rer1 cKO brain (*P* < 0.0001) (Fig 2I), but Ctip2 (Fig 2J) was not, suggesting that the generation of layer (II–IV) neurons was impaired by Rer1 loss. The neuronal progenitor marker, Tbr2 (Fig 2K) and the astrocyte marker, GFAP (Fig 2L) levels were almost unchanged in the Rer1-deficient cerebral cortex. Protein levels of BiP, a representative ER chaperone, in the Rer1-deficient cerebral cortex was slightly increased compared to that in the control mice (*P* < 0.01) (Fig 2M), suggesting that the loss of Rer1 activates ER stress in the brain to some extent. In contrast, the amount of cleaved caspase-3 levels, which indicates apoptosis rate, were almost unchanged (Fig 2N). These data suggest that the cortical malformation in the Rer1-deficient cerebral cortex occurs independently of neuronal cell death.

### Rer1 is required for neural stem cell maintenance during cortical development

**Fig 3:**
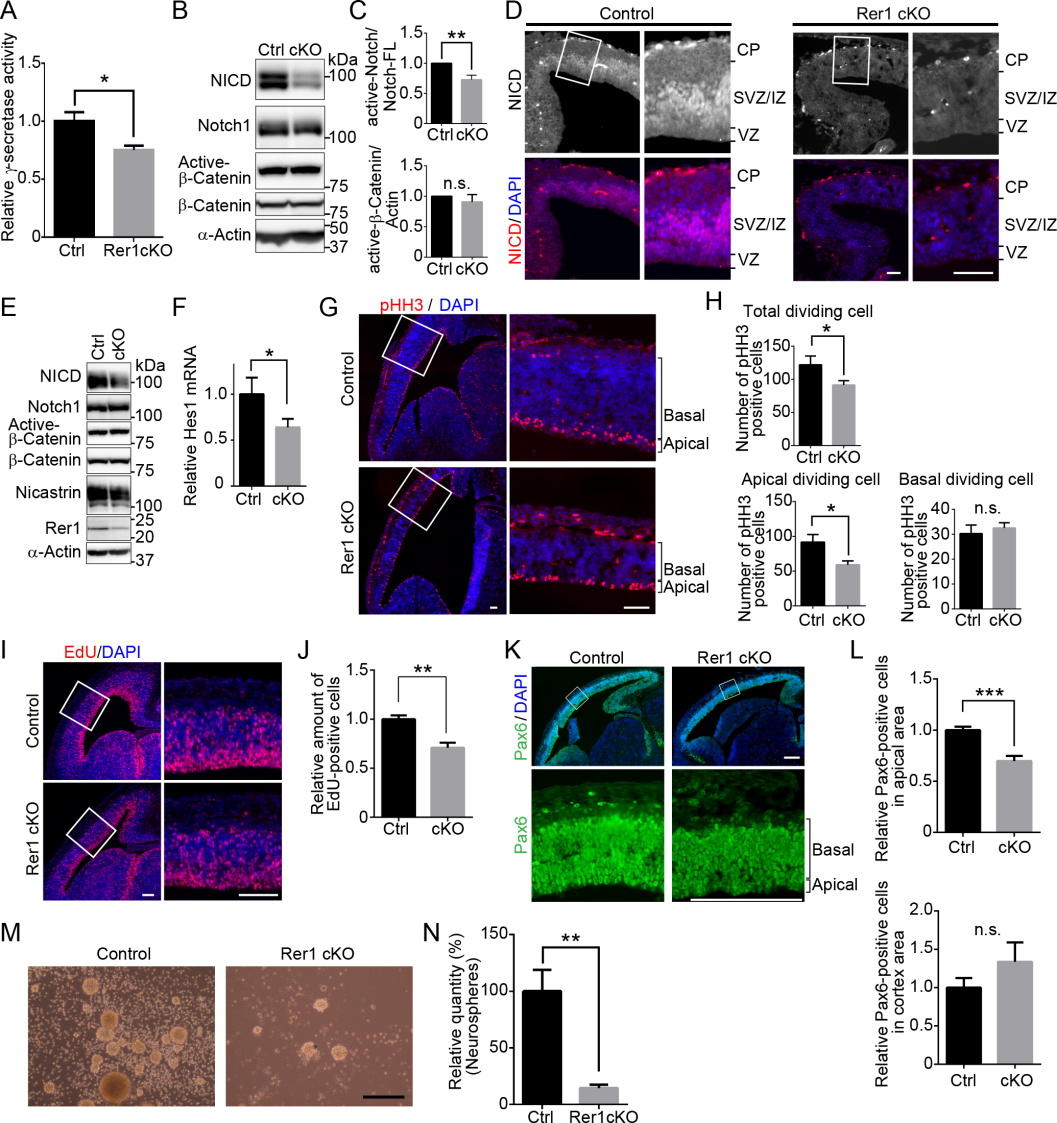
Rer1 loss reduces γ-secretase activity, leading to abnormal Notch signaling and neural stem cell proliferation. (A) Reduced γ-secretase activity in forebrain-specific Rer1 cKO mice. γ-Secretase activity was assayed in solubilized membrane fractions of cerebral cortex isolated from control and forebrain-specific Rer1 cKO mice on E18.5. Values are the mean ± standard error of the mean (SEM) of three independent experiments. The γ-secretase activities were normalized to that in control mice. *P < 0.05 (Student *t*-test). (B) Reduced release of Notch intracellular domain (NICD) on γ-secretase-dependent cleavage in forebrain-specific Rer1 cKO mice. Homogenates of the cerebral cortex dissected from control and Rer1 cKO mice on E18.5 were immunoblotted with anti-NICD, anti-Notch1, active form of β-catenin (dephosphorylated on Ser37 or Thr41), and anti-β-catenin antibodies. An anti-α-actin antibody was used as a loading control. (C) Quantification of immunoblot analysis to NICD or active-β-catenin as shown in panel B. Graphs show fold changes of values signal intensity of NICD (or active-β-catenin) normalized to that of full-length Notch1 (or α-actin) from forebrain-specific Rer1 cKO (cKO, gray bar) mice relative to that from control (Ctrl, black bar) mice. Error bars indicate the SEM for six mice. **P < 0.01 (Student *t*-test). n.s.: not significant. (D) Immunohistochemistry of NICD (red) in the cerebral cortex of control and forebrain-specific Rer1 cKO mice on E13.5. Cryosections were immunostained with an anti-NICD antibody (red). Nuclei were stained with DAPI (blue). Scale bar, 100 μm. (E) Immunoblot analysis of NICD, active-β-catenin, and NCT in control and forebrain-specific Rer1 cKO cerebral cortex on E13.5. Homogenates of the telencephalon dissected from control and forebrain specific Rer1 cKO mice on E13.5 were immunoblotted with the indicated antibodies. (F) Quantitative real-time PCR analysis of Notch target gene *Hes1* mRNA expression from control and forebrain specific Rer1 cKO brain on E13.5. Normalized data were expressed relative to the value for control mice. Error bars indicate the SEM for six mice. *P < 0.05 (Student *t*-test). (G) Immunohistochemistry for phospho-Histone H3 (pHH3) in the cerebral cortex of control and forebrain-specific Rer1 cKO mice on E13.5. Cryosections were immunostained with an anti-pHH3 antibody (red). Nuclei were stained with DAPI (blue). Scale bar, 100 μm. (H) Quantitative analysis of the number of pHH3-positive dividing cells in the cerebral cortex. The sections of cerebral cortex were prepared as shown in (G). The number of pHH3-positive cells in all area, the apical region and the basal region of the cerebral cortex in each image were counted. Data are shown as mean ± SEM of 2 sections from 5 brains from control (black bar) or forebrain-specific Rer1 cKO mice (gray bar). *P < 0.05 (Student *t*-test). n.s.: not significant. (I) EdU pulse assay of the cerebral cortex of control and forebrain-specific Rer1 cKO mice on E13.5. EdU was detected with the Click-iT EdU Alexa Fluo 594 (red). Nuclei were stained with DAPI (blue). Scale bar, 100 μm. (J) Quantitative analysis of the number of EdU-positive cells in the cerebral cortex. The number of EdU-positive cells in the cerebral cortex area in each image were enumerated. Data are shown as mean ± SEM of 10 sections from 5 brains from control (black bar) or forebrain-specific Rer1 cKO mice (gray bar). **P < 0.01 (Student *t*-test). (K) Immunohistochemistry of Pax6 in the cerebral cortex of control and forebrain-specific Rer1 cKO mice on E13.5. Cryosections were immunostained with an anti-Pax6 antibody (Green). Nuclei were stained with DAPI (blue). Scale bar, 200 μm. (L) Quantitative analysis of Pax6-positive cells in the apical area (top graph) or all cortex area (the apical and basal area, bottom graph) of the cerebral cortex in each image from forebrain-specific Rer1 cKO brain sections relative to control. Data are shown as mean ± SEM of 2 sections from 5 brains from control (black bar) or forebrain-specific Rer1 cKO mice (gray bar) on E13.5. ***P < 0.001 (Student *t*-test). n.s.: not significant. (M, N) Rer1 loss decreases the efficiency of neurosphere formation. (M) Light microscopy of neurospheres (secondary) derived from the cerebral cortex of control and forebrain specific Rer1 cKO mice on E14.5. Scale bar, 250 μm. (N) Mean values of secondary neurospheres formed by 200 dissociated cells from control and forebrain-specific Rer1cKO mice in 8 independent wells in a 96-well plate. Data were normalized to the number of neurosphere derived from the cerebral cortex from control mice. The graph shows the mean ± SEM from three independent experiments. **P < 0.01 (Student *t*-test).

Since Rer1 is involved in the γ-secretase complex formation, we investigated whether γ-secretase activity was affected in the cerebrum of forebrain-specific Rer1 cKO mice on E18.5. We examined γ-secretase activity via an *in vitro* assay, using an intramolecularly quenched fluorogenic peptide probe, which contains a C-terminal β-APP amino acid sequence cleaved by γ-secretase. We incubated membrane extracts from the cerebrum of control or forebrain-specific Rer1 cKO mice with this probe overnight at 37°C and then analyzed γ-secretase activity. In Rer1-deficient cerebrum lysates, γ-secretase activity was reduced to 80% of that in the control lysate (*P* < 0.05) (Fig 3A). We also assessed γ-secretase activity and Wnt signaling in the cerebrum by monitoring the Notch intracellular domain (NICD) and the active form of β-catenin (dephosphorylated on Ser37 or Thr41), respectively (Fig 3B). Notch is one of the known substrates of γ-secretase and plays an essential role in Notch signaling, which determines the cell fate during the development of various tissues [24, 25]. First, γ-secretase triggers Notch signaling by cleaving the intramembrane domain of Notch. The cleaved Notch intracellular domain is then translocated to the nucleus to suppress the expression of proneural genes, which regulate neural differentiation, thereby inhibiting neuronal differentiation. The level of NICD in the Rer1-deficient cerebrum was significantly decreased to 80% of that in the control lysates (*P* < 0.01) (Fig 3C). In contrast, β-catenin protein levels were comparable between E18.5 control and Rer1-deficient cerebral cortex, indicating that Wnt signaling is superficially unaffected by Rer1 loss (Fig 3B and 3C). These results suggest that Rer1 loss reduces γ-secretase activity and Notch signaling in the cerebrum. We further confirmed that Notch signaling is reduced during cerebral cortex development of Rer1-deficient mice by staining brain sections from control and forebrain-specific Rer1 cKO mice on E13.5, using an anti-NICD antibody (Fig 3D). A strong NICD signal was observed in the SVZ/intermediate zone (IZ), while a weak signal was observed in the ventricular zone (VZ) of the control cerebrum; however, the NICD signal was apparently reduced in the Rer1 cKO cerebrum. We also examined the amount of NCT, NICD, and active-β-catenin in E13.5 mouse cerebral cortex lysates from control and Rer1 cKO mice to monitor the γ-secretase complex formation, Notch signaling, and Wnt signaling, respectively (Fig 3E). NCT levels decreased in cerebral cortices of Rer1-deficient mice compared with that of control mice. Consistently, NICD levels decreased in the forebrain-specific Rer1 cKO cerebrum, suggesting that γ-secretase activity and Notch signaling are downregulated in the absence of Rer1. In contrast, active-β-catenin protein levels were comparable in the E13.5 control and Rer1-deficient cerebral cortex, indicating that Wnt signaling is unaffected upon Rer1 loss. Furthermore, *Hes1*, one of the downstream transcription factors in Notch-signaling pathway, was significantly down-regulated in the forebrain-specific Rer1 cKO mice (*P* < 0.05) (Fig 3F). Together, these results suggest that Rer1 loss decreases γ-secretase activity, thereby impairing Notch signaling, resulting in impaired cortical development in the mouse cerebrum.

Since Notch signaling plays a pivotal role in neurogenesis by regulating neural stem cell (NSC) maintenance, we investigated the population of NSCs in the Rer1-deficient cerebral cortex on E13.5, where neurogenesis occurs actively. We examined the proliferative potential of NSCs or progenitor cells via immunostaining, using an antibody against phosphorylated histone H3 (pHH3), which is highly expressed during cell division of neural stem or progenitor cells (Fig 3G). The number of pHH3-positive dividing cells significantly decreased in the Rer1-deficient cerebral cortex, especially in the apical region (*P* < 0.05) (Fig 3H). We also performed EdU incorporation assay to determine the number of proliferating cells in control and forebrain-specific Rer1 cKO cerebral cortex on E13.5 (Fig 3I). The number of proliferating cells decreased significantly in the forebrain-specific Rer1 cKO cerebral cortex relative to control mice (*P* < 0.01) (Fig 3J). Thereafter, we performed IHC, using anti-Pax6 antibody to assess the effect of Rer1 deficiency on NSCs (Fig 3K). The number of Pax6-positive cells was significantly reduced in the apical area of cerebral cortex of the forebrain-specific Rer1 cKO cerebral cortex compared to control mice, although the total number of Pax6-positive cells in the total cerebral cortex area was comparable between them (*P* < 0.001) (Fig 3L), suggesting that Rer1 is necessary for the maintenance of normal pool of neural stem cells in the ventricular zone. We further investigated whether Rer1 is required for NSC maintenance. NSCs form colonies referred to as neurospheres, *in vitro*. We harvested NSCs from the brains of control and forebrain-specific Rer1 cKO mice on E14.5 to evaluate their colony (neurosphere) formation activity from a single NSC (Fig 3M). NSCs harvested from E14.5 control mice efficiently formed neurospheres (average number of 16 colonies from 200 cells), indicating their normal self-renewal. In contrast, NSCs from Rer1-deficient mice hardly formed neurospheres (*P* < 0.01) (Fig 3N), thereby supporting the importance of Rer1 in maintaining the pool of NSCs.

### Rer1 is required for proper cell surface expression of the γ-secretase complex

**Fig 4:**
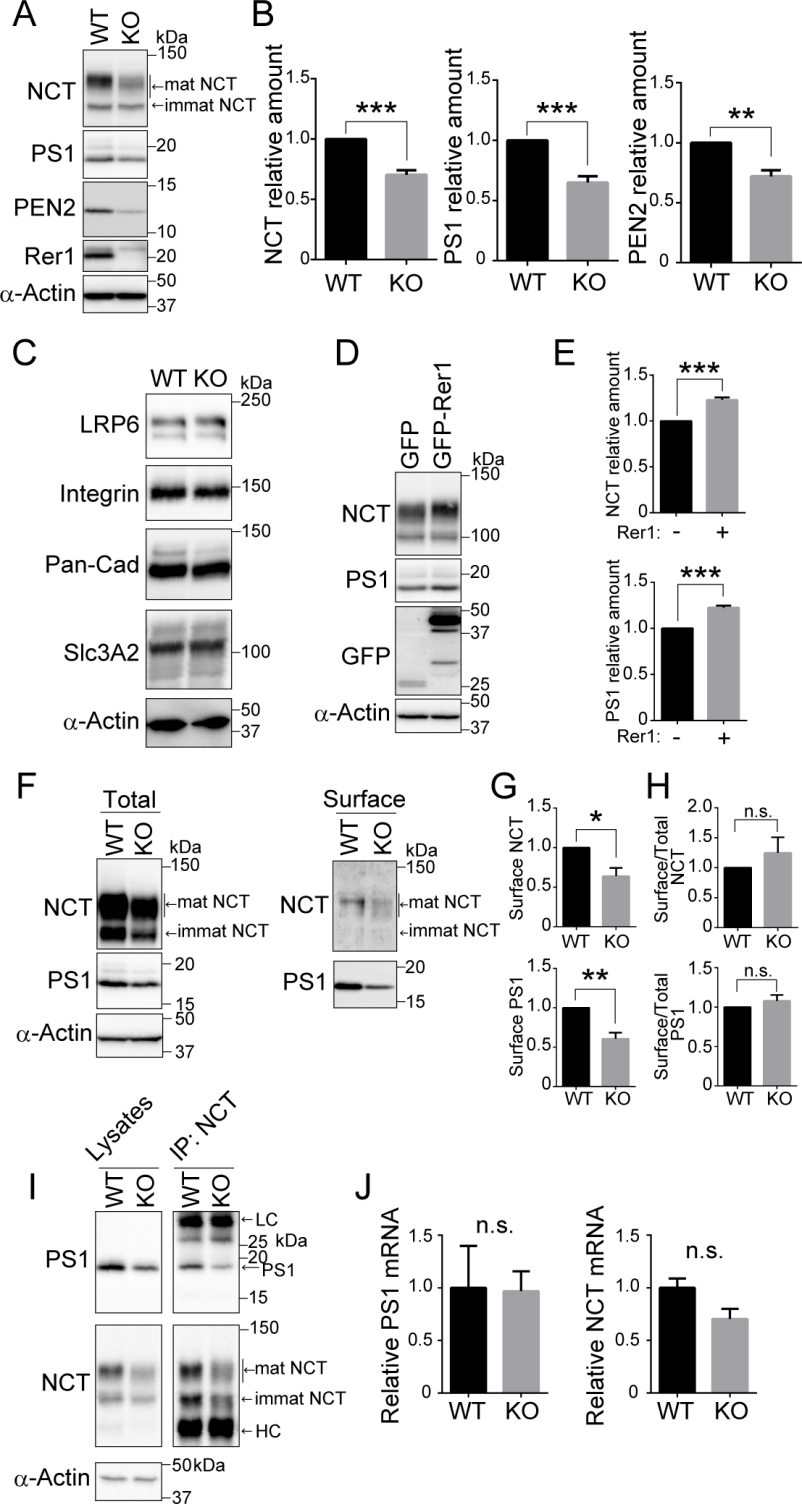
Rer1 is required for appropriate cell surface expression of γ-secretase. (A, B) Protein levels of γ-secretase components in Rer1-deficient HAP1 cells. (A) Cell lysates from wild-type (WT) or Rer1-deficient (KO) HAP1 cells were immunoblotted with the indicated antibodies. (B) Signal intensity of γ-secretase components and α-actin in each cell lysate was quantified via densitometric analysis, and the amount of each protein was normalized to the amount of α-actin. To compare the amounts of γ-secretase components in WT and Rer1-KO cells, fold changes were calculated by expressing each normalized value relative to the normalized value for each γ-secretase component in WT cells. Values are the mean ± standard error of the mean (SEM) of three independent experiments. **P < 0.01, ***P < 0.001 (Student *t*-test). (C) Protein levels of several plasma membrane proteins in Rer1-deficient cells. Cell lysates from WT or Rer1 KO HAP1 cells were immunoblotted with the indicated antibodies. (D, E) Effects of Rer1 re-expression on the levels of nicastrin (NCT) and presenilin-1 (PS1) in Rer1 KO HAP1 cells. (D) Cell lysates from Rer1 KO HAP1 cells stably expressing GFP or GFP-Rer1 were immunoblotted with the indicated antibodies. (E) Fold changes in NCT and PS1 in immunoblot analysis as shown in panel D were analyzed as described for panel A. ***P < 0.001 (Student *t*-test). (F-H) Cell surface protein biotinylation assay. (F) WT or Rer1 KO HAP1 cells were biotinylated, and biotinylated proteins were pulled down using streptavidin agarose and immunoblotted with the indicated antibodies. (G) Quantification of cell surface γ-secretase levels. The signal intensity of each γ-secretase component on the cell surface was normalized to the amount of α-actin. Fold changes for γ-secretase components relative to WT cells were analyzed as described for panel A. *P < 0.05, **P < 0.01 (Student *t*-test). n.s.: not significant. (H) Quantification of the ratio of cell surface γ-secretase to the total amount. (I) γ-secretase complex levels in WT and Rer1 KO HAP1 cells. γ-secretase complexes were immunoprecipitated from WT or Rer1 KO HAP1 cell lysates by using an anti-NCT antibody. Cell lysates (left panel) and immunoprecipitates (right panel) were immunoblotted with the indicated antibodies. LC: IgG light chain. HC: IgG heavy chain. (J) Quantitative real-time PCR analysis of *PS1* or nicastrin (*NCT*) gene expression of WT and Rer1 KO HAP1 cells. Normalized data were expressed relative to the value for WT HAP1 cells. Error bars indicate the SEM for three independent experiments. n.s.: not significant (Student *t*-test).

To investigate the effect of Rer1 depletion on γ-secretase complex formation and activity, we examined Rer1-deficient HAP1 cells generated using a CRISPR/Cas-mediated genome editing system. Immunoblot analysis using anti-Rer1 antibodies confirmed the absence of Rer1 protein in Rer1-deficient HAP1 cells (Fig 4A). Interestingly, NCT, PS1, and PEN2 protein levels in Rer1-deficient HAP1 cells decreased significantly to 60–70% of those in wild-type cells (NCT and PS1: *P* < 0.001, PEN2: *P* < 0.01) (Fig 4A and 4B). In addition, a hypoglycosylated form of NCT was observed in Rer1-deficient HAP1 cells, as reported previously [19]. We also investigated the effects of Rer1 depletion on other plasma membrane proteins such as LRP6, integrin, pan-cadherin, and Slc3A2. The levels of these proteins were unaffected in Rer1-deficient cells (Fig 4C), suggesting that the overall biosynthesis of plasma membrane proteins is not severely impaired in Rer1-deficient cells. Furthermore, we investigated whether Rer1 re-expression could recover protein levels of γ-secretase subunits in Rer1-deficient cells. We introduced GFP or GFP-Rer1 into Rer1-deficient cells, using a retroviral vector system and examined the effect of Rer1 re-expression on γ-secretase subunit levels via immunoblot analysis (Fig 4D). The expression of GFP-Rer1 in Rer1-deficient cells increased NCT and PS1 levels by 1.2 folds compared with that in control cells expressing GFP alone (*P* < 0.001) (Fig 4E). We also examined the cell surface levels of NCT and PS1 in wild-type and Rer1-deficient cells via a cell surface biotinylation assay (Fig 4F). We labeled cell surface proteins with biotin and pulled them down using avidin beads to detect γ-secretase levels at the plasma membrane. Immunoblot analysis indicated that the amount of NCT and PS1 on the cell surface of Rer1-deficient cells was reduced to approximately 60% of that in wild-type cells (NCT: *P* < 0.05, PS1: *P* < 0.01) (Fig 4G). However, the ratio of cell surface NCT and PS1 to total proteins was comparable in wild-type and Rer1-deficient cells (Fig 4H). To investigate whether NCT assembles with PS1 in Rer1-deficient cells, we immunoprecipitated γ-secretase complexes from wild-type and Rer1-deficient cell lysates, using an anti-NCT antibody and examined the immunoprecipitates upon immunoblotting with antibodies against PS1 and NCT (Fig 4I). The association of NCT and PS1 was detected in Rer1-deficient cells, suggesting that γ-secretase subunits can form a complex even in the absence of Rer1. We also examined the gene expression of γ-secretase components via quantitative PCR analysis. The expression of PS1 and NCT in Rer1-KO HAP1 cells was comparable to that in control cells, suggesting that gene expression of γ-secretase components was not largely affected by the loss of Rer1 (Fig 4J).

### Rer1 loss results in the degradation of γ-secretase subunits

**Fig 5:**
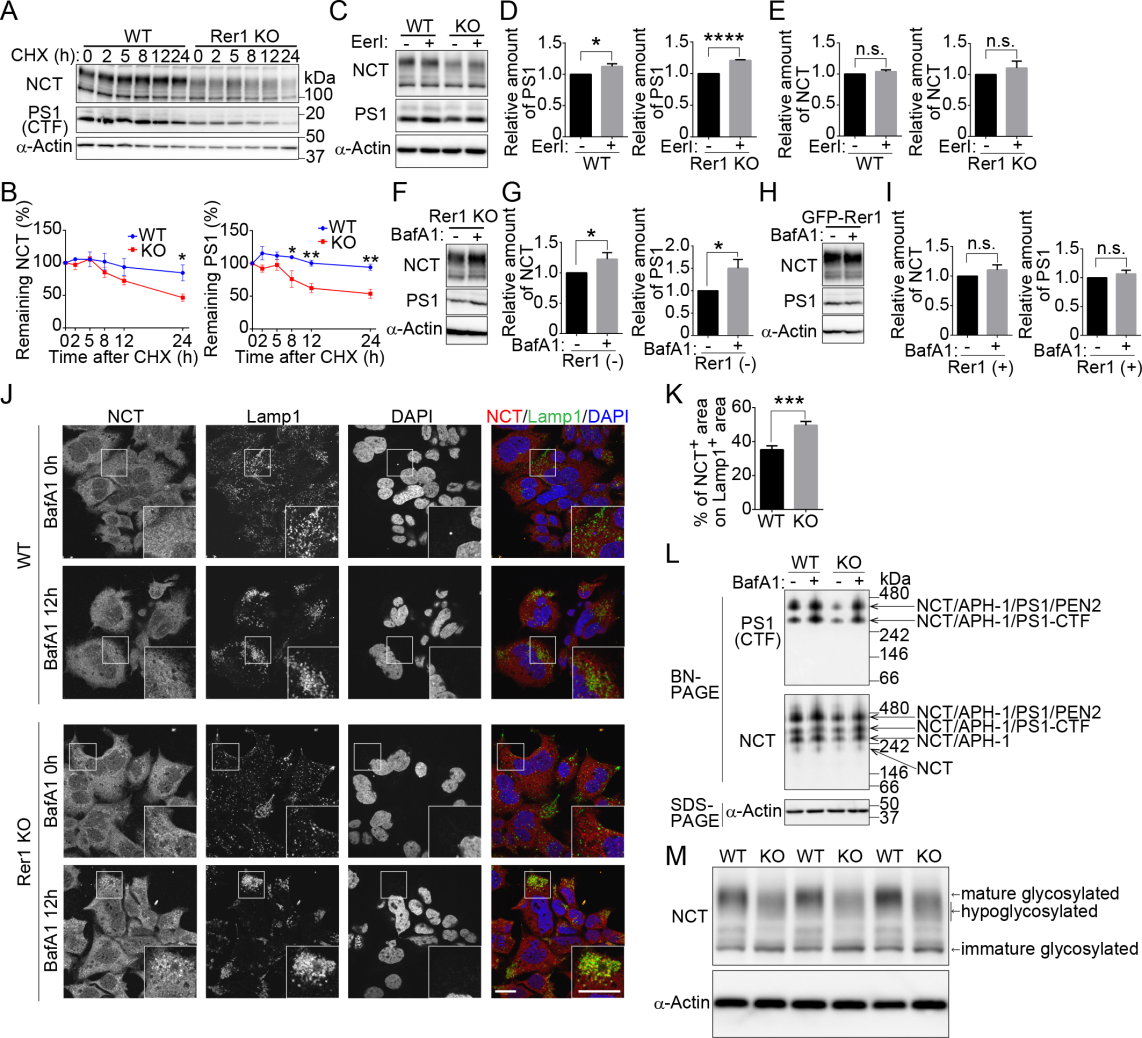
Rer1 deficiency leads to the degradation of nicastrin and presenilin-1 in lysosomes. (A) Cycloheximide (CHX) chase experiments for nicastrin (NCT) and presenilin-1 C-terminal fragment (PS1 CTF) in WT or Rer1-deficient (KO) HAP1 cells. HAP1 cells were treated with 10 μg/ml CHX, and protein levels of NCT, PS1 CTF, and α-actin were measured at the indicated times. Cell lysates were immunoblotted with the indicated antibodies. (B) Quantitative analysis of NCT and PS1 CTF during CHX chase experiments. Graphs show relative levels of NCT and PS1 CTF normalized to α-actin. Values are the mean ± standard error of the mean (SEM) of three independent experiments. Two-way analysis of variance (ANOVA) was performed to determine the significance of the differences. *P < 0.05, **P < 0.01 (ANOVA). (C) Effects of ERAD inhibitor on the stability of NCT and PS1 CTF in WT or Rer1 KO HAP1 cells. WT and Rer1 KO HAP1 cells were cultured for 16 h in the presence (+) or absence (-) of 2.5 μM eeyarestatin I (EerI). Cell lysates were immunoblotted with the indicated antibodies. (D, E) Quantitative analysis of the effects of ERAD on PS1 CTF (D) and NCT (E) in WT and Rer1 KO cells. Graphs show fold changes for γ-secretase components in cells treated with EerI (+) relative to those in vehicle (DMSO)-treated cells (-). Values are the mean ± SEM of three independent experiments. *P < 0.05, ****P < 0.0001 (Student *t*-test). n.s.: not significant. Note that the total amount of NCT and PS1 CTF are reduced in Rer1 KO HAP1 cells. (F-I) Lysosomal degradation of NCT and PS1 CTF in Rer1 KO HAP1 cells. (F) Rer1 KO HAP1 cells were cultured for 16 h in the presence (+) or absence (-) of 200 nM bafilomycin A1 (BafA1). Cell lysates were immunoblotted with the indicated antibodies. (G) Quantitative analysis of the effects of BafA1 on NCT and PS1 CTF. Graphs show fold changes of NCT and PS1 CTF in cells treated with BafA1 (+) relative to vehicle (DMSO) (-) treated cells. Values are the mean ± SEM of three independent experiments as shown in panel F. *P < 0.05 (Student *t*-test). (H) Immunoblot analysis of Rer1 KO cells expressing GFP-Rer1 with the indicated antibodies. Cells were treated as shown in Fig 5F (I) Quantification of immunoblot experiments as shown in panel H. (J) Accumulation of NCT in lysosomes in BafA1-treated Rer1 KO cells. WT and Rer1 KO cells were cultured for 12 h in presence (+) or absence (-) of BafA1 and immunostained using anti-NCT (red) and anti-Lamp1 (green) antibodies. Nuclei were visualized with DAPI (blue). Immunostained cells were observed using confocal laser-scanning microscopy. Scale bar, 20 μm. (K) Quantitative analysis of colocalization between NCT- and Lamp1-positive area. The percentage of NCT-positive area on Lamp1-positive area was calculated using cellSens software (Olympus). The graph shows the average colocalization percentage per cell. Error bars represent the SEM (n = 58 and n = 46 of WT and Rer1 KO HAP1 cells, respectively). ***P < 0.001 (Student *t*-test). (L) γ-Secretase complex formation in WT and Rer1 KO HAP1 cells. WT and Rer1 KO HAP1 cells were treated with or without 200 nM BafA1 for 24 h. Cell lysates were subjected to Blue-Native (BN-PAGE) and SDS-PAGE analysis and were immunoblotted with the indicated antibodies. (M) Underglycosylation of NCT in Rer1 KO HAP1 cells. WT or Rer1 KO HAP1 cells were immunoblotted with the indicated antibodies.

To investigate the role of Rer1 in the stability of the γ-secretase complex, we examined the turnover rates of NCT and PS1 in wild-type and Rer1-deficient cells via treatment with a protein synthesis inhibitor, cycloheximide (CHX). The γ-secretase complex is known to be formed by sequential assembly of APH-1, NCT, PS1 and PEN2 in order [26]. PS1 is then processed endoproteolytically to a 28-kDa N-terminal fragment and a 17-kDa C-terminal fragment [27]. We used an anti-PS1 antibody, which can detect a C-terminal fragment of PS1 (PS1 CTF). Full-length PS1 is hardly detected at a steady state because it is quickly degraded via a proteasome-dependent mechanism or endoproteolysis [26, 28], whereas PS1 CTF stably exists in a complex. NCT and PS1 CTF largely remained in wild-type cells at 24 h after CHX treatment (Fig 5A). By contrast, levels of both proteins started to decrease at 5 h after CHX treatment in Rer1-deficient cells. Approximately 50% of NCT and PS1 were degraded within 24 h in Rer1-deficient cells compared with wild-type cells, in which most of both proteins remained at 24 h (Fig 5B).

Furthermore, we investigated whether γ-secretase subunits were degraded by the proteasome or lysosomes in Rer1-deficient cells. We treated wild-type and Rer1-deficient cells with a potent inhibitor of ER-associated protein degradation (ERAD), eeyarestatin I (EerI), or a proteasome inhibitor, MG132, and investigated the involvement of the proteasome in the degradation of γ-secretase subunits (Fig 5C and S4 Fig). ERAD inhibition significantly increased PS1 CTF levels in wild-type and Rer1-deficient cells (*P* < 0.05 and *P* < 0.0001, respectively), as reported previously [28] (Fig 5D). NCT levels also tended to increase slightly but not significantly in the presence of EerI (Fig 5E), suggesting that PS1 CTF and NCT are partially degraded by the proteasome in wild-type and Rer1-deficient cells. Since γ-secretase subunits began to decrease 5 h after CHX treatment in Rer1-deficient cells, we assumed that NCT and PS1 CTF might be partly transported to and degraded in lysosomes. To test this hypothesis, we treated Rer1-deficient cells with bafilomycin A1 (BafA1), which blocks lysosomal degradation by inhibiting V-ATPase. NCT and PS1 CTF protein levels significantly increased in BafA1-treated Rer1-deficient cells compared with that in vehicle (DMSO)-treated cells (Fig 5F and 5G). The effects of BafA1 on NCT and PS1 CTF accumulation were alleviated in GFP-Rer1-expressing Rer1-deficient cells (Fig 5H and 5I). Furthermore, we investigated whether some γ-secretase subunits are transported to lysosomes in Rer1-deficient cells. We incubated wild-type and Rer1-deficient cells with or without BafA1 and observed the subcellular localization of NCT via immunostaining with antibodies against NCT and a lysosomal marker Lamp1 (Fig 5J). Endogenous NCT mainly localized to the ER in a meshwork pattern and partly to punctate structures in wild-type cells treated with or without BafA1. Lamp1-positive lysosomes tended to aggregate in a cellular region in HAP1 cell lines in the presence of BafA1. NCT-positive puncta subtly increased in Rer1-deficient cells at a steady state and prominently increased in Rer1-deficient cells treated with BafA1. Approximately 50% of NCT-positive puncta localized close to the Lamp1-positive lysosomes or overlapped with them in BafA1-treated Rer1-deficient cells (Fig 5K), indicating that NCT is partly transported to lysosomes. These results suggest that immature γ-secretase subunits or complexes are no longer retained in the ER and are mislocalized to lysosomes for degradation in the absence of Rer1. We also examined γ-secretase complex formation via Blue Native PAGE (BN-PAGE) (Fig 5L). The amount of γ-secretase complex was drastically reduced in Rer1-KO cells compared to that in control cells; however, complex formation still occurred to some extent under this condition. Notably, the reduction in γ-secretase complex was recovered upon addition of BafA1, which inhibits lysosomal function, suggesting that γ-secretase subunits are largely targeted to and degraded in lysosomes in the absence of Rer1. Furthermore, NCT was underglycosylated in Rer1-deficient cells, as reported previously [19], in Rer1-knockdown cells (Fig 5M), suggesting that Rer1 is required for the cell surface expression of fully matured γ-secretase complex.

## Discussion

The present study shows that Rer1 exerts positive effects in the maintenance of γ-secretase activity by mediating proper expression of γ-secretase complex on the cell surface. Additionally, Rer1 is involved in the proliferation of NSCs during cerebral cortex development by modulating the onset of Notch signaling, which regulates cortical development and higher brain function. These findings suggest that Rer1 functions as the early-Golgi quality control, which mediates normal expression and functioning of the γ-secretase complex during mammalian brain development.

Rer1 has been proposed as a limiting factor, which negatively regulates the assembly of γ-secretase subunits, since siRNA-mediated knockdown of Rer1 facilitates γ-secretase assembly and cell surface expression in cultured cells [19]. However, we observed that γ-secretase activity in the brain lysates was significantly decreased in Rer1-deficient mice. In addition, we found that a complete loss of Rer1 significantly reduced γ-secretase components on the cell surface probably because of their mistargeting to lysosomes for degradation. These results raise a question regarding the different effects of Rer1 knockdown and Rer1 deletion on expression of the γ-secretase complex. In both cases, some of the γ-secretase subunits or complexes are transported to post-Golgi compartments similarly both in Rer1-knockdown and knockout cells; however, their destinations are different. One possible explanation is that late-Golgi or post-Golgi quality control systems function more strictly in HAP1 cells and mice than in other cultured cell lines used in previous studies and deliver immature γ-secretase subunits or complexes to lysosomes. Alternatively, a small amount of Rer1 in Rer1-knockdown cells might be sufficient to support the maturation of γ-secretase subunits or complexes, allowing delivery to the cell surface. By contrast, a prolonged and profound lack of Rer1 may allow for leakage of γ-secretase subunits or complexes from the ER, resulting in their lysosomal targeting.

Our observations suggest that Rer1 exerts positive effects on the cell surface expression of γ-secretase. In yeast, Rer1p is required for accurate formation of an iron transporter complex consisting of Fet3p and Ftr1p by retaining unassembled Fet3p in the ER [7]. In the absence of Rer1p, unassembled Fet3p is mislocalized to vacuoles and degraded there. Ftr1p also becomes unstable and degraded probably by ER-associated degradation in Rer1-deficient cells, suggesting a positive role of Rer1p in the formation of the iron transporter complex. Furthermore, Rer1 loss leads to lysosomal degradation of unassembled components such as the γ-subunit of acetylcholine receptor and immature rhodopsin in mammalian cells [6, 22]. These observations support our hypothesis that Rer1 mediates the assembly or maturation of membrane protein complexes as the early-Golgi quality control system by retaining unassembled components until they form an appropriately matured complex. Interestingly, some of the γ-secretase components and/or complex were targeted to lysosomes for degradation in Rer1-deficient cells, suggesting the existence of late-Golgi or post-Golgi quality control system [4, 29, 30]. Multiple quality control systems through the exocytosis pathway would allow for efficient formation of membrane protein complexes and their cell surface expression at a proper timing.

NSCs harvested from Rer1-deficient cerebral cortex hardly form neurospheres, indicating that self-renewal of NSCs is partly impaired by Rer1 loss. In addition, Rer1 deficiency in the developing mouse cerebrum decreased the number of mature neurons, including Cux1-positive upper layer neurons in the cortex, and reduced the size of the cerebral cortex (Figs 1–3). In such mice, the number of dividing NSCs decreased in the cerebral cortex. These results suggest that Rer1 loss impairs the proliferation of NSCs, which finally leads to the exhaustion of neuronal progenitor cells.

Interestingly, these phenotypes of Rer1-deficient mice are reminiscent of those of mice defective in γ-secretase and Notch signaling [31–33]. γ-Secretase triggers Notch signaling, which is known to regulate the expansion of the neuronal progenitor pool for appropriate regulation of brain size. In this study, we demonstrated that Notch signaling was significantly reduced in the Rer1-deficient cerebrum probably because of a reduced activity of γ-secretase complex. These findings suggest that Rer1 maintains Notch signaling by supporting proper γ-secretase complex expression to supply a sufficient progenitor pool for cortical development. Recently, it has been reported that Rer1 is also involved in the assembly and transport of voltage-gated sodium channels in Purkinje cell [34]. Thus, Rer1 could also affect other membrane protein complexes such as voltage-gated sodium channels or the activity of other γ-secretase substrates in addition to Notch1 to regulate brain development and behaviors.

In humans, *Rer1*, located in 1p36 region, is significantly associated with 1p36-deletion syndrome. 1p36-deletion syndrome is caused by a large deletion in the subtelomeric region of chromosome 1, and it occurs in 1 in 5000 people. Patients with this syndrome exhibit a brain disorder with mental retardation and behavioral disorder. In addition, they also have structural brain abnormalities, including a large anterior fontanelle and microcephaly. Notably, Rer1 loss in the cerebral cortex resulted in cerebral hypoplasia and behavioral disorder (Fig 1), which recapitulates the brain defects present in most patients with 1p36-deletion syndrome, suggesting that Rer1 mutations are one of the causative factors for this syndrome. However, the symptoms in patients result from haploinsufficiency due to 1p36 microdeletion. Since Rer1 heterozygous mice did not develop the striking phenotypic abnormalities observed in forebrain-specific Rer1-deficient mice, heterozygous deletion of the other genes would also contribute to brain anomalies observed in 1p36-deletion syndrome. Interestingly, the downstream targets of Notch signaling such as Hes4 and Hes5 are also present in this region [35], although over 90 genes are contained in the 1p36 microdeletion. Reduced expression of such genes together with Rer1 in patients may cause abnormal brain development. Further investigation of γ-secretase activity and Notch signaling in patients with 1p36-deletion syndrome will be important for establishing the involvement of Rer1 and the pathogenic mechanisms underlying the disease etiology.

## Methods

### Ethics statement

All animal procedures were performed in accordance with the guidelines of the Animal Care and Experimentation Committee of Gunma University, and all animals were bred at the Institute of Animal Experimental Research of Gunma University.

### Gene trap mice

FlipRosaβgeo-trapped embryonic stem (ES) cell clones no. EUCJ0172c09 (EUCOMM) were used to generate heterozygous mice (*Rer1*^*+/trap*^), using standard protocols (S1 and S2 Figs).

### Generation of tissue-specific Rer1-deficient mice

The FlipRosaβgeo cassette comprises a conventional gene trap element and pairs of inversely oriented heterotypic recombinase target sites (RTs), such as *loxP* and *FRT* sites that flank the gene trap element (S3 Fig). FLPe and Cre recombinases can invert the gene trap element of FlipRosaβgeo flanked by RTs via directional site-specific recombination, thereby first repairing and then re-inducing the gene trap mutation [36]. To generate the mouse (*Rer1*^*+/inv-trap*^) harboring FLPe-inverted gene trap insertions, in which the gene trap mutation for *Rer1* expression is invalidated, we crossed *Rer1*^*+/trap*^ mice with *actin-flippase* transgenic mice (B6; SJL-TG (ACTFLPe) 9250Dym/J) (Jackson Laboratory). To generate forebrain-specific *Rer1*-deficient mice, *Emx1-Cre* transgenic mice [23] were crossed with the *Rer1*^*+/inv-trap*^ mice to re-induce the gene trap mutation via Cre recombinase-mediated inversion in the forebrain.

### Cell culture and stable expression

Rer1-deficient HAP1 cell lines, edited by CRISPR/Cas to contain a frame shift mutation in a coding exon 3 of Rer1, were obtained from horizon discovery (Horizon Discovery). The guide RNA sequence was as follows: 5’-CACCCTACACGGCTGTGCGA-3’. HAP1 cells were cultured in Iscove’s Modified Dulbecco’s Medium (IMDM) supplemented with 10% fetal calf serum and penicillin/streptomycin (Wako) in a 5% CO_2_ incubator at 37°C. HAP1 cells expressing GFP or GFP-Rer1 were generated via retroviral transduction. To generate retroviruses, Plat-E cells were co-transfected with pMXs-IP-GFP-mouse Rer1 and pCG-VSV-G using FuGENE HD (Promega). HAP1 cells were then infected with the recombinant retroviruses and selected in medium containing 3 μg/mL puromycin, as reported previously [37].

### Reagents

MG132, γ-secretase inhibitor (L685, 458), and a fluorescence-quenching substrate for γ-secretase (Nma-Gly-Gly-Val-Val-Ile-Ala-Thr-Val-Lys(Dnp)-D-Arg-D-Arg-D-Arg-NH_2_) were purchased from the Peptide Institute, Japan. Protease inhibitor cocktail (complete EDTA-free protease inhibitor) and eeyarestatin I were purchased from Roche, USA. Bafilomycin A1 was from Wako Pure Chemical, Japan; cycloheximide from MP Biomedicals, USA; CHAPSO from Dojin, Japan; and Sulfo-NHS-Biotin from Thermo Fisher Scientific, USA.

### γ-Secretase assay

E18.5 mouse cerebral cortices were homogenized using Dounce homogenizer in homogenate buffer (20 mM HEPES, pH 7.5, 50 mM KCl, 2 mM EGTA) with a protease inhibitor cocktail. Lysates were centrifuged at 4°C, 800 × *g* for 10 min. The resulting supernatants were centrifuged at 4°C, 100,000 × *g* for 1 h. The membrane pellet was resuspended in a buffer containing 20 mM HEPES pH 7.0, 150 mM KCl, 2 mM EGTA, 1% CHAPSO, and protease inhibitor cocktail. γ-Secretase activity was measured by incubating solubilized membranes with the fluorescence-quenching substrate for γ-secretase overnight at 37°C in the absence or presence of L-685, 458, as reported previously [38].

### Immunoblot analysis

Mouse brains were homogenized in ice-cold homogenate buffer (50 mM Tris-HCl pH 7.4, 0.25 M sucrose, 1 mM EDTA) with a protease inhibitor cocktail, using the mini homogenizer tube BioMasher II (Nippi). After homogenization, an equal amount of lysis buffer (50 mM Tris-HCl pH 7.4, 250 mM NaCl, 1% Triton X-100, 0.1% SDS, 0.2% sodium deoxycholate, and 1 mM EDTA) was added and the lysates were incubated on ice for 30 min. The lysates were centrifuged at 4°C, 15,000 rpm for 15 min. Supernatants were subjected to immunoblot analysis using specific antibodies. HAP1 cells were lysed in a cell lysis buffer (50 mM Tris-HCl pH 7.4, 0.2% SDS, 1% sodium deoxycholate) with a protease inhibitor cocktail and 1 mM phenylmethanesulfonyl fluoride (PMSF). The lysates were centrifuged at 4°C, 15,000 rpm for 15 min and the supernatants were then subjected to immunoblot analysis using specific antibodies.

### Immunofluorescence staining

HAP1 cells were cultured on coverslips and fixed with 3% paraformaldehyde in PBS for 10 min. The cells were permeabilized with 0.1% Triton X-100 and incubated with PBS containing 5% NDS for 1 h for blocking, and then treated with specific antibodies. Images were acquired using an FV1000 confocal microscope (Olympus) with a 100× PlanApo oil immersion lens (1.40 numerical aperture; Olympus).

### Antibodies

The following primary antibodies were used for immunoblotting: anti-RER1 (Sigma, R4407), anti-Nicastrin (Sigma, N1660), anti-Presenilin-1 [D39D1] (Cell Signaling Technologies, 5643), anti-Presenilin-1 [PS1loop] (Millipore, MAB5232), anti-PEN2 (Cell Signaling Technologies, 5451), anti-CD49b (BD, 611016), anti-LRP6 (Cell Signaling Technologies, 2560), anti-Pan-cadherin (Cell Signaling Technologies, 4068), anti-CD98 (Santa Cruz, sc-9160), anti-GFP (Fitzgerald, RDI-GRNFP3abg), anti-actin [C4] (Millipore, MAB1501), anti-NeuN [A60] (Millipore, MAB377), anti-GFAP (Frontier Institute, GFAP-Rb-Af800), anti-Cux1 (Santa Cruz, sc13024), anti-Ctip2 (Abcam, ab18465), anti-Tbr2 (Abcam, ab23345), anti-NICD [D3B8] (Cell Signaling Technologies, 4147), anti-Notch1 [D1E11] (Cell Signaling Technologies, 3608), anti-β-catenin (BD, 610153), anti-active-β-catenin [8E7] (MERCK, 05–665), and anti-cleaved caspase-3 (Cell Signaling Technology, 9661) antibodies. The following primary antibodies were used for immunostaining: anti-Nicastrin (Sigma, N1660), anti-Lamp1 [H4A3] (Santa Cruz, sc20011), anti-phospho-Histone H3 [Ser10] (Cell Signaling Technologies, 9701), anti-GFAP (Frontier Institute, GFAP-Rb-Af800), anti-Cux1 (Santa Cruz, sc13024), anti-Ctip2 (Abcam, ab18465), anti-Tbr2 (Abcam, ab23345), anti-Pax6 (BioLegend, PRB-278P) and anti-NICD [D3B8] (Cell Signaling Technologies, 4147) antibodies. The following secondary antibodies were used for immunostaining: goat anti-rabbit Alexa Fluor-568, goat anti-mouse Alexa Fluor-647, donkey anti-rabbit Alexa Fluor-594, and donkey anti-rat Alexa Fluor-594 (all from Life Technologies) antibodies. Rabbit anti-Rer1 antibodies were generated against the C-terminal regions of mouse Rer1 (NH_2_-C+KRRYKGKEDVGKTFAS-COOH coupled to KLH; TK craft corp.).

### EdU labeling and detection

Pregnant female mice were intraperitioneally administered 50 mg kg^-1^ body weight of EdU. After 2 h, E13.5 embryos were fixed in 4% paraformaldehyde (PFA) in phosphate-buffered saline (PBS). Subsequently, brain tissues were immersed in 15% and then 30% sucrose in PBS and frozen for sectioning. EdU detection was performed in accordance with the manufactures’ Click-it EdU Alexa 594 imaging kit protocol (Thermo Fisher Scientific).

### Immunohistochemical analysis

Adult mice were transcardially perfused with 4% paraformaldehyde in phosphate buffer (pH 7.4), as described previously [39]. Brain tissues from embryos were fixed in the same fixative solution overnight. Thereafter, tissues were embedded in paraffin, sectioned, and then stained using Meyer's H&E. For immunohistochemistry for paraffin-embedded tissue, tissue sections were subjected to antigen retrieval with a microwave oven in 0.01 M citrate buffer (pH 6.0) for 10 min. After blocking with 3% H_2_O_2_ in MeOH for 30 min and then 5% BSA in PBS for 30 min, sections were incubated with primary antibodies, followed by incubation with EnVision Plus System-HRP Labelled Polymer Anti-Rabbit kit (K4003, DakoCytomation). The signal was visualized using Liquid DAB substrate chromogen system (DakoCytomation). Hematoxylin was used as a counterstain. For immunohistochemical analysis of cryosections, fixed tissues were cryoprotected via sequential immersion in 15% and 30% sucrose in PBS overnight and were embedded in Tissue-Tek OCT compound (Sakura Finetek). After embedding, 10-μm cryosections were cut and immunolabeled. The tissue sections were then subjected to antigen retrieval in Histo VT One (NACALAI TESQUE) at 70°C for 10 min (for pHH3 and Pax6 staining), target retrieval solution S1700 (DakoCytomation) at 105°C for 15 min (for NICD staining), target retrieval solution S1700 (DakoCytomation) at 90°C for 5 min (for Cux1 staining), and 0.01 M citrate buffer (pH 6.0) at 90°C for 10 min (for Tbr2, Ctip2, and GFAP staining). After blocking with 5% BSA in PBS for 30 min, sections were incubated with primary antibodies, followed by incubation with fluorescently labeled secondary antibodies. The stained sections were analyzed via confocal microscopy (FV1000, Olympus) or fluorescence microscopy (BZ-9000, Keyence). GFAP-, Pax6-, or EdU-positive cells were enumerated using BZ-X analyzer software (Keyence).

### Behavioral analysis

Behavioral tests were performed with male 3-month-old littermates. The open-field test and rotarod test were performed, as described previously [40]. All tests used equipment from O’Hara & Co. Ltd.

### Neuronal stem cell culture

Neural cells were isolated from E14.5 cerebral cortex. The isolated cells were plated at a density of 2 × 10^5^ cells/mL in KBM Neural Stem Cell (KOHJIN BIO #16050100) with KBM neural stem cell supplement containing epidermal growth factor and fibroblast growth factor (KOHJIN BIO) and cultured at 37°C in 5% CO_2_. After 1 week, neurospheres were collected and dissociated by TrypLE (ThermoFisher Scientific), and 200 dissociated cells were plated in each well of a 96-well ultra-low attachment plate (Corning) for secondary sphere formation. The secondary neurospheres were enumerated after 1 week of culture.

### Cell surface labeling

Cells were washed with ice-cold HBSS twice (Gibco) and incubated for 30 min on ice with 0.5 mg/mL Sulfo-NHS-Biotin (Thermo Fisher Scientific) diluted in PBS. After three washes with HBSS, cells were incubated with 50 mM NH_4_Cl for 5 min to quench excess biotin. Cells were then lysed with a lysis buffer (50 mM Tris-HCl pH 7.5, 150 mM NaCl, 0.5 mM EDTA, 1% Triton X-100, Protease Inhibitor Cocktail). Cell lysates were pulled down with streptavidin agarose resin (Thermo Fisher Scientific) overnight. Pulled down samples were washed twice with lysis buffer and then subjected to immunoblot analysis, using specific antibodies.

### Blue Native PAGE

HAP1 cells were prepared with a native sample buffer (Thermo Fisher Scientific), containing 0.5% DDM (*n*-dodecyl β-ᴅ-maltoside) and a protease inhibitor mixture and subjected to Blue Native PAGE, using the Novex Bis-Tris gel system (Thermo Fisher Scientific) in accordance with the manufacturer’s instructions.

### Quantitative real-time PCR analysis

Total RNA was extracted using the RNeasy mini kit (Qiagen) and subjected to RT with a ReverTra Ace qPCR RT kit (Toyobo). The resulting cDNA was then subjected to real-time PCR analysis with 1 x SYBR Green PCR master mix (Takara) and gene specific primers. Assays were performed with Thermal Cycler Dice Real Time System III (Takara). The sequence of the various primers (the forward primer and the reverse primer, respectively) were 5′-GGACCCGAGAAGACCTCCTT-3′ and 5′-GCACATCACTCAGAATTTCAATGG-3′ for mouse acidic ribosomal phosphoprotein, 5′-TATCATGGAGAAGAGGCGAAGG-3′ and 5′-TTCTCTAGCTTGGAATGCCGG-3′ for mouse *Hes1*, 5′-GAAGGTGAAGGTCGGAGTCA-3′ and 5′-TGGACTCCACGACGTACTCA-3′ for human *GAPDH*, 5′-AGCAGTATCCTCGCTGGTGA-3′ and 5′-TGAAATCTCCCAATCCAAGTTT-3′ for human *PSEN1*, 5′-CAGTGGCTTCCTTTGTCACC-3′ and 5′-GAGCTGCCAATGTAGTCAAAAG-3′ for human *NCSTN*.

### Cycloheximide (CHX) chase experiment

HAP1 cells were treated with 10 μg/mL CHX, and the cell extracts were prepared at specified time points and subjected to immunoblot analysis with the indicated antibodies.

### Eeyarestatin I, MG132, or bafilomycin A1 (BafA1) treatment for immunoblot analysis of PS1 CTF and NCT

HAP1 cells were treated with 5 μM MG132 for 2 h, 2.5 μM eeyarestatin I, or 200 nM BafA1 for 16 h. Cell lysates were then subjected to immunoblot analysis with indicated antibodies.

### Statistical analysis

Data were analyzed using GraphPad Prism 6 (GraphPad Software, Inc., USA), and expressed as mean ± standard error of the mean values. Two-tailed Student’s *t*-tests and two-way analysis of variance was used to evaluate significance and calculate *P* values. *P* values less than 0.05 were considered statistically significant.

## Supporting information

S1 Fig Generation of mice harboring the inserted gene trap cassette at the *Rer1* locus. (A) Schematic diagrams of the *Rer1* gene trap allele. The gene trap vector, which consists of splice acceptor site linked to a β-geo selectable marker, was inserted into the 1^st^ intron of the *Rer1* gene on chromosome 4. Exon 2 encodes the translation initiation codon of the Rer1 protein. The indicated probe containing the neomycin fragment in PGK-Neo-polyA hybridizes with a 5.2kb EcoRI fragment from the inserted allele. PCR primers to identify the gene-trap vector are indicated. (B) Validation of gene trap cassette insertion in ES cells. PCR using each primer set illustrated in panel A confirmed the insertion of gene trap vector in *Rer1* locus. (C) Southern blotting of the trap vector insertion in *Rer1*^*+/trap*^ mice. Genomic DNA from *Rer1*^*+/+*^ and *Rer1*^*+/trap*^ mice tails was digested with EcoRI for hybridization with the probe in panel A. (D) Summary of genotyping analysis of staged embryos from *Rer1*^+/trap^ intercrosses. No alive *Rer1*^*trap/trap*^ embryo after E9.5 or pup was detected in the uterus or after birth.
(TIF)

S2 Fig Growth defects in *Rer1*^*+/trap*^ mouse. (A) Representative images of a control (*Rer1*^*+/+*^) and a *Rer1*^*+/trap*^ littermate at 12 weeks (male) and 7 week (female) of age. Rer1 heterozygous mice had a small body size. (B) Relative weight of control (*Rer1*^*+/+*^) and *Rer1*^*+/trap*^ littermates at 4 weeks of age. (C) Tissue blot analysis of control (*Rer1*^*+/+*^) and *Rer1*^*+/trap*^ littermates. Tissue homogenates from (*Rer1*^*+/+*^) and *Rer1*^*+/trap*^ mice were immunoblotted with an anti-Rer1 antibody. An anti-α-tubulin antibody was used as a loading control.
(TIF)

S3 Fig Conditional inactivation of Rer1 expression by the recombinase-mediated inversion of gene trap cassette. (A) Schematic representation of conditional gene inactivation by FlipRosaβgeo. FLPe induces the inversion of the SAβgeo gene trap cassette onto the antisense at either FRT or F3 sequences. The simultaneously inverted F3 (in case of the inversion at FRT) or FRT (in case of the inversion at F3) site is excised, and thereby the cassette is locked against reinversion. Cre recombinase reinverts the SAβgeo cassette onto the sense at either loxP or lox511. FRT (yellow triangles) and F3 (green triangles), heterotypic target sequences for the FLPe recombinase; loxP (red triangles) and lox511 (pink triangles), heterotypic target sequences for the Cre recombinase; SA, splice acceptor; βgeo, β-galactosidase/neomycin phosphotransferase fusion gene; pA, bovine growth hormone polyadenylation sequence. Primer positions within FlipRosaβgeo are indicated. (B) Validation of gene trap cassette inversion. PCR using primer sets illustrated in panel A confirmed the inversion of gene trap vector in *Rer1* locus. Nestin-Cre mice were used to express Cre recombinase in the brain. (C) Cre-mediated inactivation of Rer1 expression in *Rer1*^*trap/trap*^ mouse embryonic fibroblasts (MEFs). MEFs with indicated genotypes were infected with adenovirus expressing Cre recombinase (Ad-Cre) and Rer1 protein level was examined by immunoblot analysis.
(TIF)

S4 Fig Effects of a proteasome inhibitor on the stability of NCT and PS1 CTF in Rer1 KO HAP1 cells. (A) Rer1 KO HAP1 cells transfected with (+) or without (-) GFP-Rer1 using a retrovirus vector were cultured for 2 h in the presence (+) or absence (-) of 5 μM MG132. Cell lysates were immunoblotted with the indicated antibodies. (B, C) Quantitative analysis of the effects of MG132 on NCT (B) and PS1 CTF (C) in Rer1 KO cells (-) and Rer1 KO cells stably expressing GFP-Rer1 (+). Graphs show fold changes for γ-secretase components in cells treated with MG132 (+) relative to those in vehicle (DMSO)-treated cells (-). Values are the mean ± SEM of three independent experiments. ***P < 0.001 (Student *t*-test). n.s.: not significant. Note that the total amounts of NCT and PS1 CTF are reduced in Rer1 KO HAP1 cells.
(TIF)

S1 Table Numerical data. (XLSX)
