# Supplementary figures and images for: Rer1-mediated quality control system is required for neural stem cell maintenance during cerebral cortex development
Source: PLoS Genet. 2018 Sep 27;14(9):e1007647. doi: 10.1371/journal.pgen.1007647 (PMC6159856; doi:10.1371/journal.pgen.1007647)

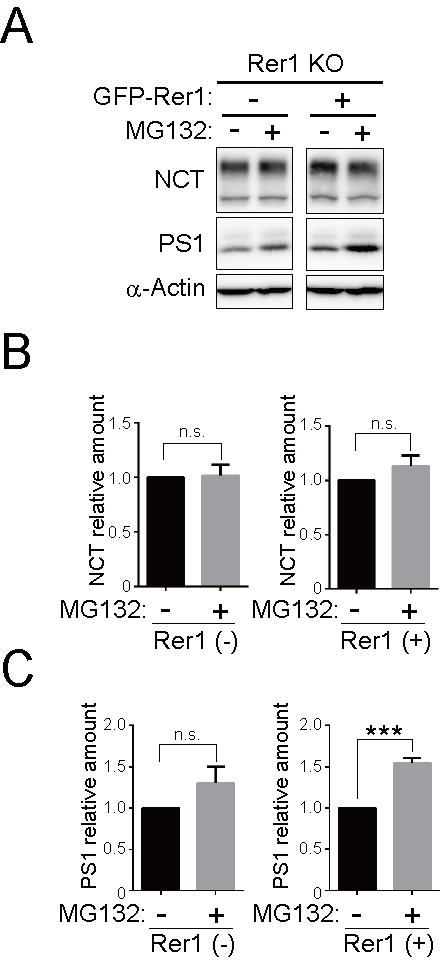

Supplement: S4 Fig — (A) Rer1 KO HAP1 cells transfected with (+) or without (-) GFP-Rer1 using a retrovirus vector were cultured for 2 h in the presence (+) or absence (-) of 5 μM MG132. Cell lysates were immunoblotted with the indicated antibodies. (B, C) Quantitative analysis of the effects of MG132 on NCT (B) and PS1 CTF (C) in Rer1 KO cells (-) and Rer1 KO cells stably expressing GFP-Rer1 (+). Graphs show fold changes for γ-secretase components in cells treated with MG132 (+) relative to those in vehicle (DMSO)-treated cells (-). Values are the mean ± SEM of three independent experiments. ***P < 0.001 (Student t-test). n.s.: not significant. Note that the total amounts of NCT and PS1 CTF are reduced in Rer1 KO HAP1 cells. (TIF) [file pgen.1007647.s004.tif]
